# A prediction model using machine-learning algorithm for assessing intrathecal hyperbaric bupivacaine dose during cesarean section

**DOI:** 10.1186/s12871-021-01331-8

**Published:** 2021-04-14

**Authors:** Chang-na Wei, Li-ying Wang, Xiang-yang Chang, Qing-he Zhou

**Affiliations:** 1 Department of Anesthesia, Jiaxing University Affiliated Women and Children Hospital, Jiaxing, Zhejiang Province China; 2 Department of Anesthesia, Affiliated Hospital of Jiaxing University, Jiaxing, Zhejiang Province China

**Keywords:** Machine learning algorithm, Bupivacaine dosage, Cesarean section, Spinal anesthesia, Physical variables

## Abstract

**Background:**

The intrathecal hyperbaric bupivacaine dosage for cesarean section is difficult to predetermine. This study aimed to develop a decision-support model using a machine-learning algorithm for assessing intrathecal hyperbaric bupivacaine dose based on physical variables during cesarean section.

**Methods:**

Term parturients presenting for elective cesarean section under spinal anaesthesia were enrolled. Spinal anesthesia was performed at the L3/4 interspace with 0.5% hyperbaric bupivacaine at dosages determined by the anesthesiologist. A spinal spread level between T4-T6 was considered the appropriate block level. We used a machine-learning algorithm to identify relevant parameters. The dataset was split into derivation (80%) and validation (20%) cohorts. A decision-support model was developed for obtaining the regression equation between optimized intrathecal 0.5% hyperbaric bupivacaine volume and physical variables.

**Results:**

A total of 684 parturients were included, of whom 516 (75.44%) and 168 (24.56%) had block levels between T4 and T6, and less than T6 or higher than T4, respectively. The appropriate block level rate was 75.44%, with the mean bupivacaine volume [1.965, 95%CI (1.945,1.984)]ml. In lasso regression, based on the principle of predicting a reasonable dose of intrathecal bupivacaine with fewer physical variables, the model is “Y=0.5922+ 0.055117* X_1_-0.017599*X_2_” (Y: bupivacaine volume; X_1_: vertebral column length; X_2_: abdominal girth), with λ 0.055, MSE 0.0087, and R^2^ 0.807.

**Conclusions:**

After applying a machine-learning algorithm, we developed a decision model with R^2^ 0.8070 and MSE due to error 0.0087 using abdominal girth and vertebral column length for predicting the optimized intrathecal 0.5% hyperbaric bupivacaine dosage during term cesarean sections.

**Supplementary Information:**

The online version contains supplementary material available at 10.1186/s12871-021-01331-8.

## Background

**Cesarean** delivery is routinely performed under spinal anesthesia with hyperbaric bupivacaine [1–3]. Bupivacaine provides an appropriate duration of anesthesia to perform cesarean delivery, and hyperbaric bupivacaine may ensure a more predictable block [4]. However, it is still a challenge for the anesthetist to achieve the optimal spinal spread for cesarean delivery [5]. Parturient variables, including age, height, weight, and body mass index (BMI) have been used to predict spinal spread, but results have been inconsistent [6, 7].

Previous studies demonstrated that abdominal girth and vertebral column length correlated favorably with spread of intrathecal bupivacaine in term parturients [8, 9]. Furthermore, an elemental regression equation was established between parturient vertebral column length, abdominal girth, and 0.5% hyperbaric intrathecal bupivacaine volume for T5 block level [10]. However, the sample size in these studies was relatively small and the accuracy of the regression equation needed further verification [8, 10].

In recent years, there has been an advance in machine-learning algorithms in several fields including anesthesiology, which allowed large amounts of data for the development of robust predictive analytics [11–14]. These were was used to predict, interalia, postinduction hypotension [14], intraoperative hypotension [12], and length of hospital stay [15]. In our hospital, more than 1000 parturient women undergo cesarean section under spinal anesthesia annually. The purpose of this prospective study was to develop a more precise decision-support model based on more sample size using a machine-learning algorithm for assessing intrathecal hyperbaric bupivacaine dosage based on their physical variables at cesarean section.

## Methods

### Patients

The study protocol was approved by the Ethical Committee of Jiaxing Maternity and Child Health Care Hospital in May 2015 (2015–5) and pre-registered at http://www.chictr.org.cn/ index.aspx (ChiCTR-OOC-16009149) on September 3, 2016. The signed informed consent was obtained from all participants. In this prospective observational study, 684 term parturient women who presented for elective cesarean section under spinal anesthesia were enrolled from October 2016 to November 2019. Inclusion criteria were American Society of Anesthesiologists (ASA) physical status II or III, age between 21 and 40 years, and gestational age over 37 completed weeks. Exclusion criteria were patient refusal, significant medical or obstetric comorbidities, multiple pregnancies, failed spinal anesthesia, contraindication to spinal anesthesia, discrepancy in spread between both midclavicular lines, or history of allergy to bupivacaine.

### Spinal anaesthesia

The parturient fasted for 8 h and received 8 mL/kg Ringer’s lactate solution via peripheral intravenous access before spinal anesthesia. After entry to the operation room, the standard ASA monitoring was performed. Before spinal puncture, the L3/4 interspace was confirmed by ultrasonic imaging. Spinal anesthesia was performed at the L3/4 interspace in the left lateral decubitus position using a 25 G pencil-point spinal needle (Zhejiang Sujia Medical Equipment Co., Ltd) with midline approach; 0.5% hyperbaric bupivacaine (Shanghai Hefeng Pharmaceutical Co., Ltd) was injected into the subarachnoid space over 10 s after a free flow of cerebrospinal fluid had been obtained. The volume of 0.5% hyperbaric bupivacaine administered was based on the experience of the anesthesiologist. After these procedures, the woman was then rapidly placed in a supine position, with a right pelvic wedge placed to facilitate left uterine displacement. All anesthesia procedures were performed by the same attending physician, and the assessment of cephalad spread of spinal anesthesia was completed by another anesthetist who was blind to the measurement information about the woman. The spinal spread was assessed in both midclavicular lines by an 18-gauge needle for loss of pinprick discrimination 15 min after intrathecal injection and then surgery commenced. If the parturient complained of severe pain (general Visual Analogue Scale [VAS] ≥ 4) during surgery, remifentanil was administered with micropump injection for rescue analgesia. If, based on the assessment of the anesthetist, the woman could not endure the surgery under spinal anesthesia, general anesthesia was performed. Hypotension was defined as systolic pressure values < 90 mmHg or systolic pressure decreases > 30% and was treated with 6 mg of ephedrine or 100 μg of phenylephrine intravenously. Bradycardia was defined as heart rate values < 55 beats/min and was treated with atropine 0.5 mg intravenously.

### Outcome measures

Abdominal girth was measured at the level of the umbilicus at the end of expiration when the parturient was placed supinely on the horizontal operating table. Vertebral column length was measured from the C7 vertebra to the sacral hiatus (C7-SH). Cephalad spread of spinal anesthesia was assessed by testing for loss of pinprick discrimination in both midclavicular lines at 3 min intervals after intrathecal injection. The spread level at 15 min after intrathecal bupivacaine injection was used for the analysis. The spinal spread level between T4-T6 was considered the appropriate block level. The volume of intrathecal bupivacaine was recorded. Parturient demographic variables, including age, height, fundal height, and weight were recorded. Demographic variables, such as fetal biparietal diameter, were also recorded.

As predictors for the machine-learning models, we included parturient age, height, fundal height, weight, abdominal girth, vertebral column length, and fetal biparietal diameter. The primary outcome was to obtain the regression equation between optimized intrathecal 0.5% hyperbaric bupivacaine dosage and physical variables.

### Decision-support model analysis

Machine-learning algorithm was performed by Python 3.7.1. The objective function of lasso regression is:

$$ \underset{\omega }{\min }{\left\Vert y- X\omega \right\Vert}_2^2+\lambda {\left\Vert \omega \right\Vert}_1 $$

In the function, λ is a parameter that controls the complexity of the model. With lasso regression, controlling the parameter of λ can result in a sparse solution: the coefficient of unimportant features will be assigned to 0, and the important features will be highlighted in order according to the weight value so as to achieve the importance ranking of features, and further control the complexity of the algorithm according to the selected input features.

Therefore, based on the lasso regression algorithm, our study explored the relationship between the seven individual physical variables listed above and bupivacaine dosage, then obtained the regression equation between optimized intrathecal 0.5% hyperbaric bupivacaine dosage and the physical variables.

The statistical model strives to predict an intrathecal bupivacaine dosage with fewer physical variables. R^2^ is the determination coefficient, which is the degree of fitting for the obtained regression equation. Mean-square error (MSE) is a single value that provides information about the goodness fit of the regression line. The smaller the MSE value, the better the fit, as smaller values imply smaller magnitudes of error. The data set (term parturient with appropriate block level) was split into derivation (80%) and validation (20%) cohorts. The derivation cohort parturients were used to derive for the prediction model, and the validation cohorts were used to validate the model. We gradually increased the number of independent variables from 2 to 5 (physical variables), optimized the λ value, and balanced the value of R^2^, MES, and the number of independent variables.

All statistical analyses were performed with SPSS version 19 (IBM Corp). We presented the data as means ± standard deviations (SDs) or numbers (%) as appropriate. We tested quantitative data by using the independent *t-*test. A two-tailed *p* value < 0.05 denoted statistical significance.

## Results

A total of 684 parturients were included in this study, of whom 516 had a block level between T4 and T6 (appropriate block level) and 168 had a block level lower than T6 or higher than T4 (inappropriate block level). The appropriate block level rate was 75.44%, with the mean bupivacaine volume [1.965, 95%CI(1.945,1.984)]ml. The spread level of 69 parturients was less than T6 and 99 parturients higher than T4. Compared with those with appropriate block levels, weight and abdominal girth were greater in the group with inappropriate block levels (*p* < 0.05 and *p* < 0.01, respectively); there was no obvious difference in bupivacaine dosage and other physical variables in those women with appropriate and inappropriate block levels (Table 1).

**Table 1:** Clinical characteristics of term parturients

	Overall	Appropriate block	Inappropriate block	*P* value
No. pts. (%)	684	516 (75.44%)	168 (24.56%)	–
Age, yrs	30.7 ± 4.5	30.7 ± 4.5	30.8 ± 4.6	0.717
Weight, kg	69.5 ± 9.6	69.1 ± 9.4	71.0 ± 10.1	0.022
Height, cm	159.3 ± 4.7	159.4 ± 4.6	159.1 ± 4.8	0.405
Vertebral column length, cm	56.6 ± 3.2	56.7 ± 3.0	56.4 ± 3.8	0.323
Abdominal girth, cm	99.9 ± 7.1	99.5 ± 6.9	101.2 ± 7.7	0.007
Fundal height, cm	35.2 ± 2.8	35.2 ± 2.9	35.2 ± 2.6	0.789
Fetal biparietal diameter, cm	9.1 ± 0.5	9.1 ± 0.5	9.2 ± 0.4	0.076
Fetal weight, kg	3.3 ± 0.7	3.4 ± 0.7	3.4 ± 0.6	0.445
Bupivacaine dosage, mL	2.0 ± 0.2	2.0 ± 0.2	2.0 ± 0.1	0.057

A total of 412 term parturients were used to derive for the prediction model. In lasso regression, when the physical variables included in the equation increased from 2 to 5, R^2^ increased from 0.8070 to 0.81325, and MSE decreased from 0.0087 to 0.00844, both with little changed.

**Fig. 1:**
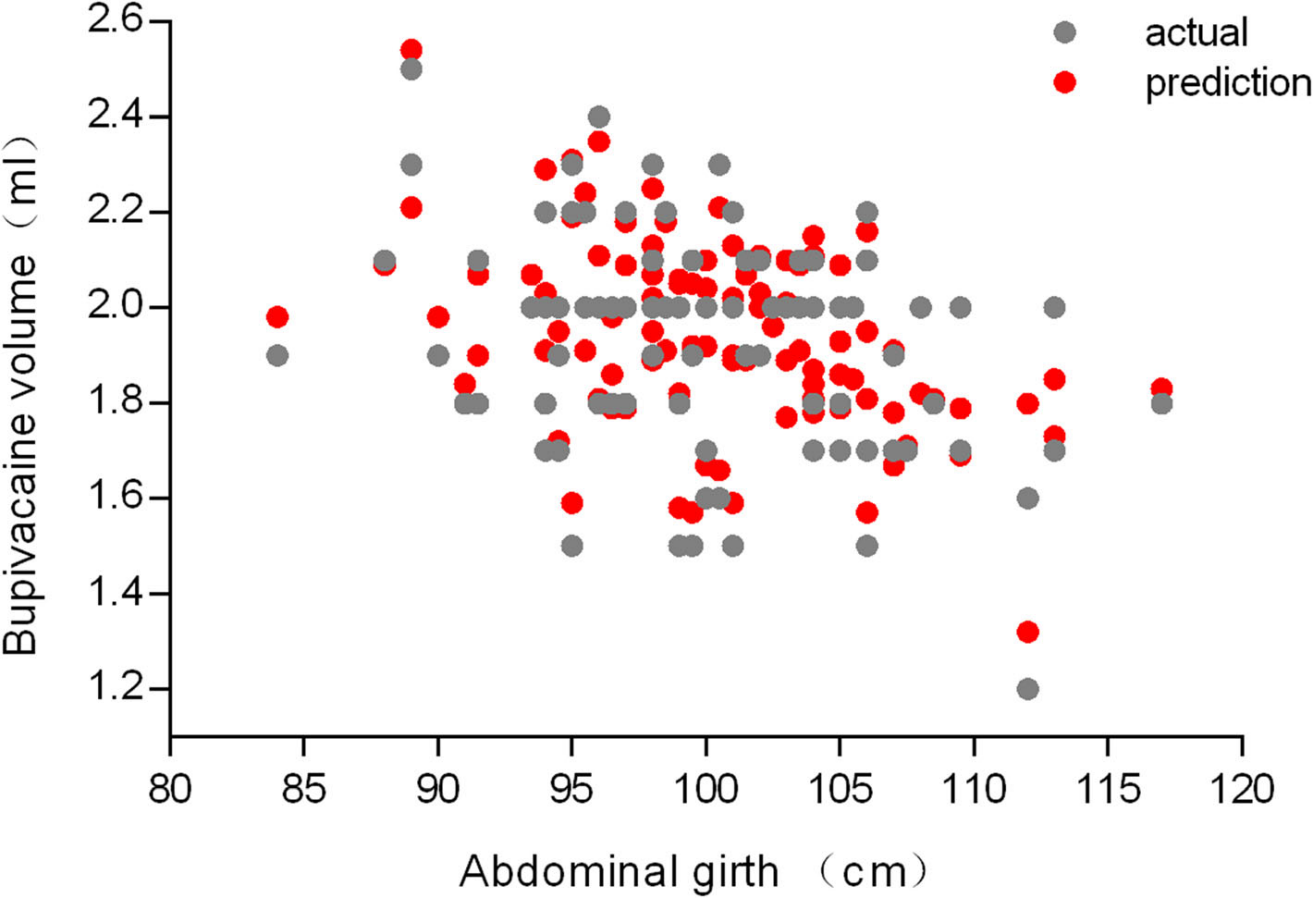
Actual and predicted bupivacaine volume for validation cohorts with 104 term parturients for validation; the ordinate is bupivacaine volume and abscissa is abdominal girth

**Fig. 2:**
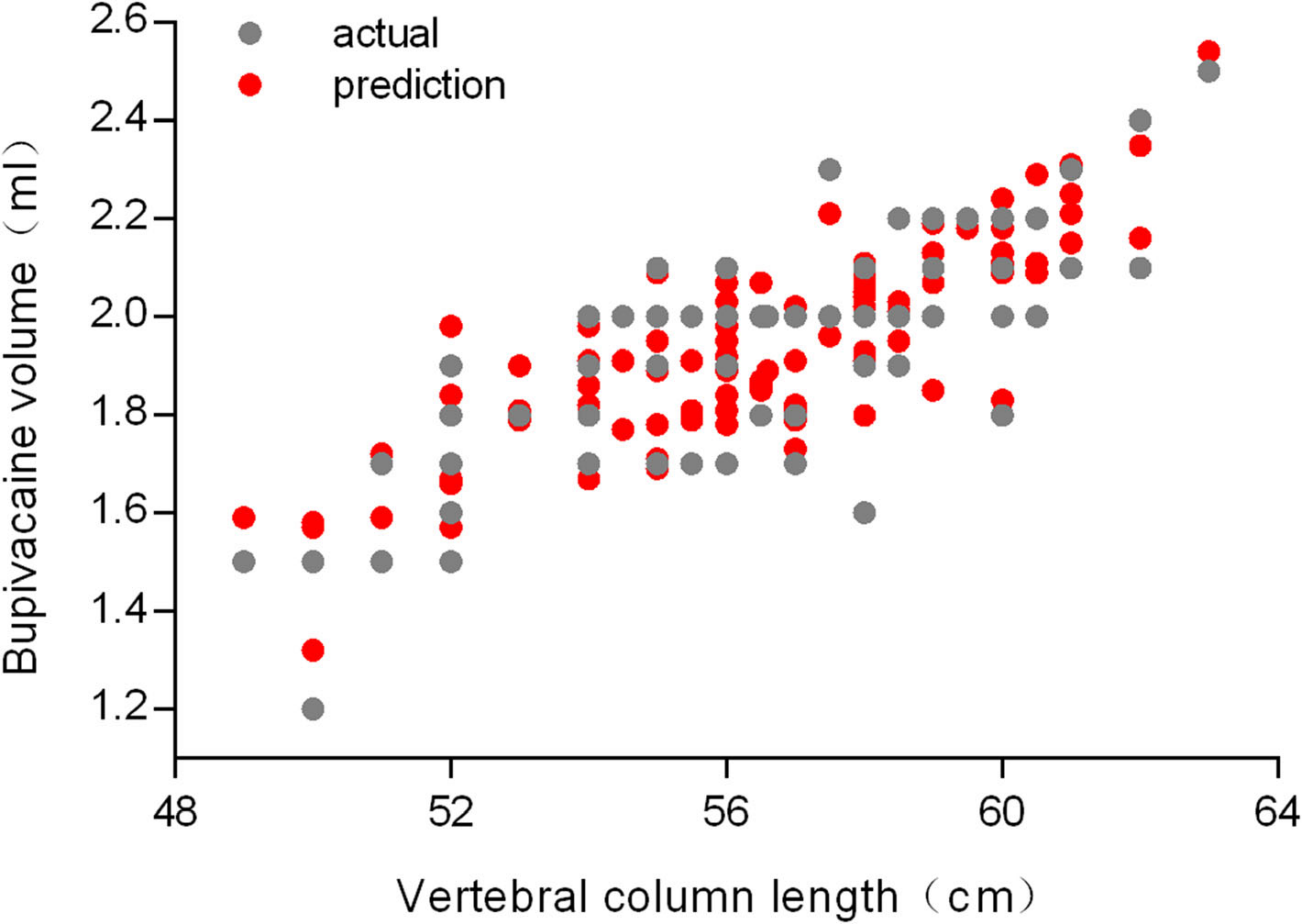
Actual and predicted bupivacaine volume for validation cohorts with 104 term parturients for validation; the ordinate is bupivacaine volume and abscissa is vertebral column length

On decision tree analysis, vertebral column length and abdominal girth were the top two physical performance variables with respect to the dosage of intrathecal bupivacaine; the model is “Y=0.5922+ 0.055117* X_1_-0.017599* X_2_”(Y: 0.5% bupivacaine volume; X_1_: vertebral column length; X_2_:abdominal girth), with the λ 0.055, MSE 0.0087 and R^2^ 0.807 (Table 2). The remaining 104 participants were used to validate the model. The actual bupivacaine volume was 1.94 ± 0.21 mL, with a predicted bupivacaine volume of 1.95 ± 0.19 mL (*p* > 0.05); R^2^ of all women during the validation was above 0.8, indicating that the model was reliable (Figs. 1 and 2).

**Table 2:** Parameter selection of lasso regression and model evaluation for different parameters

λ Value	Selected physical variable	Weight for variable	Equation Intercept	MSE	R^2^
0.055	Abdominal girth	-0.017599	0.5922	0.0087	0.8070
	Vertebral column length	0.055117			
0.038	Height	0.000141	0.5071	0.0086	0.8108
	Abdominal girth	− 0.018174			
	Vertebral column length	0.057225			
0.031	Height	0.000286	0.4516	0.0085	0.81199
	Weight	−0.000171			
	Abdominal girth	−0.018232			
	Vertebral column length	0.058107			
0.020	Height	0.000618	0.3687	0.00844	0.81325
	Weight	−0.000600			
	Fundal height	−0.001253			
	Abdominal girth	−0.017910			
	Vertebral column length	0.059371			

*MSE* Mean-square error, *R*^*2*^ Coefficient of determination

In the current study, 282 parturients (41.23%) were found to develop hypotension during cesarean section and were treated with ephedrine and/or norepinephrine.

## Discussion

In this prospective, observational study, after applying a machine-learning algorithm, we developed the decision model: Y = 0.5922 + 0.055117* X_1_–0.017599* X_2_ (Y: 0.5% hyperbaric bupivacaine volume; X_1_: vertebral column length; X_2_: abdominal girth), with the λ 0.055, MSE 0.0087, and R^2^ 0.807. On decision-tree analysis, vertebral column length and abdominal girth were the top two performance physical variables with respect to intrathecal bupivacaine dosage.

According to the data type of this study, multiple linear regression, ridge regression, and lasso regression are usually used for data analysis. Multiple linear regression has the characteristics of simple construction, easy implementation, and low operation complexity, but the model is prone to overfitting. To control the complexity of the model, penalties or other constraints are often added to the model; that is, regularization techniques. Ridge regression and lasso regression are currently popular regularization regression techniques. The goal of the two is the same; that is, to minimize the sum of squared residuals, but the constraints on the regression coefficients are different. Ridge regression can effectively solve the problem of overfitting but, as the value changes, its feature coefficients become uniformly small, making it impossible to discern the importance of each feature. Lasso regression, however, effectively solves the overfitting problem; it can obtain a sparse solution by controlling the parameters: the coefficients of unimportant features will be assigned to 0, and the important features will be highlighted in order according to the weight value, so as to achieve the importance ranking of features, and further control the complexity of the algorithm according to the selected input features.

In the present study, according to the results of parameter selection of lasso regression and model evaluation for different parameters, when the physical variables included in the equation increased from 2 to 5, MSE and R^2^ were not obviously increased. On decision-tree analysis, vertebral column length and abdominal girth were the top two performance physical variables with respect to intrathecal bupivacaine dosage. Over one hundred term parturients were used to validate the model, and R^2^ of all patients during the validation was above 0.8, indicating that the model was reliable.

Previous studies reported that the median satisfactory block height for the loss of pinprick discrimination during spinal anesthesia for cesarean section was T5, and the interquartile range (IQR) was from T4-T6 [16]. In our previous study, we set T5 as the appropriate spinal spread level and found the vertebral column length and abdominal girth to be the top two performance physical variables [10]. In the current study, we set appropriate spinal spread levels for the loss of pinprick discrimination as T6, T5, and T4 at 15 min after intrathecal injection, and also found vertebral column length and abdominal girth to be the top two performance physical variables. We need to note that the regression model obtained in this study is more clinically valuable.

In current study, the appropriate block level rate was 75.44%, with the mean bupivacaine volume 1.965 ml (9.825 mg). The mean bupivacaine dose was slightly lower than previous study reported that bupivacaine provides anesthesia in almost all patients (ED95) at doses that range between 11 and 13 mg [17]. It may be that the study subjects only included Asians.

Studies have proven that, in pregnant women, soft tissues may migrate inward into the vertebral canal [18], and engorgement of the extradural venous plexus occurs when pregnant women are in the supine position because of obstruction of the inferior vena cava by the enlarged uterus [19, 20]. Thus, an increased abdominal girth in the parturient causes a decrease in lumbosacral cerebrospinal fluid volume. Carpenter et al. [21]. reported that lumbosacral cerebrospinal fluid volume is the main determinant of the spread of spinal anesthesia. Our recent study showed that abdominal girth and dorsosacral distance were correlated with lumbosacral cerebrospinal fluid volume [22]. Therefore, the maternal abdominal girth and vertebral length may have a predictive effect on the spinal spread due to their effect on the volume of the lumbosacral cerebrospinal fluid.

Previous studies reported incidences of hypotension varying from 1.9 to 71% [23, 24]. In the present study, hypotension during cesarean section occurred in 41.23% of participants and was treated with ephedrine and/or norepinephrine. Most of these patients were term parturients with appropriate spinal spread levels. Thus, when performing a cesarean section under spinal anesthesia, we must strictly monitor the patient’s hemodynamic status, irrespective of whether or not the anesthesia block level is in the appropriate range.

This study has several limitations. First, only the spread level at 15 min after intrathecal bupivacaine injection was used for the analysis. We know that the spinal spread changes over time. Second, intrathecal hyperbaric bupivacaine with opioid was not studied in current study and it is worthy of further study. Third, the model does not take into account the multitude of other factors, such as drug factors, position factors, surgical factors and so on. Forth, this model is based on Asians, and whether it is accurate in other races requires further research. Despite these limitations, the current machine-learning algorithm provides new insights into the potential impact of controversial parameters.

## Conclusion

In conclusion, in the current study, after applying a machine-learning algorithm, we developed a decision model with the coefficient of determination 0.807 and the mean of squares due to error 0.0087, using two physical variables for predicting the intrathecal 0.5% hyperbaric bupivacaine dosage during cesarean section in term parturients. Among the parturient physical variables, abdominal girth and vertebral column length were the two most significant factors, which can be used for predicting the intrathecal bupivacaine dosage during cesarean section.

## Supplementary Information

**Additional file 1: Figure S1.** Visual analysis diagram of correlation between physical variables and intrathecal bupivacaine dose for the parturients with block levels between T4 and T6. **Figure S2.** Correlation analysis diagram between physical variables and intrathecal bupivacaine dose for the parturients with block levels between T4 and T6.

## Data Availability

The datasets used and/or analyzed during the current study are available from the corresponding author on reasonable request (Email: jxxmxy@163.com).

## Abbreviations

MSE: Mean-square Error
R^2^: Coefficient of determination
BMI: body mass index
ASA: American Society of Anesthesiologists
VAS: Visual Analogue Scale
SPSS: Statistic Package for Social Science
IQR: Inter quartile range
