# Supplementary material for: A prediction model using machine-learning algorithm for assessing intrathecal hyperbaric bupivacaine dose during cesarean section
Source: BMC Anesthesiol. 2021 Apr 14;21:116. doi: 10.1186/s12871-021-01331-8 (PMC8045295; doi:10.1186/s12871-021-01331-8)

Fig 1. Visual analysis diagram of correlation between physical variables and intrathecal bupivacaine dose for the parturients with block levels between T4 and T6.

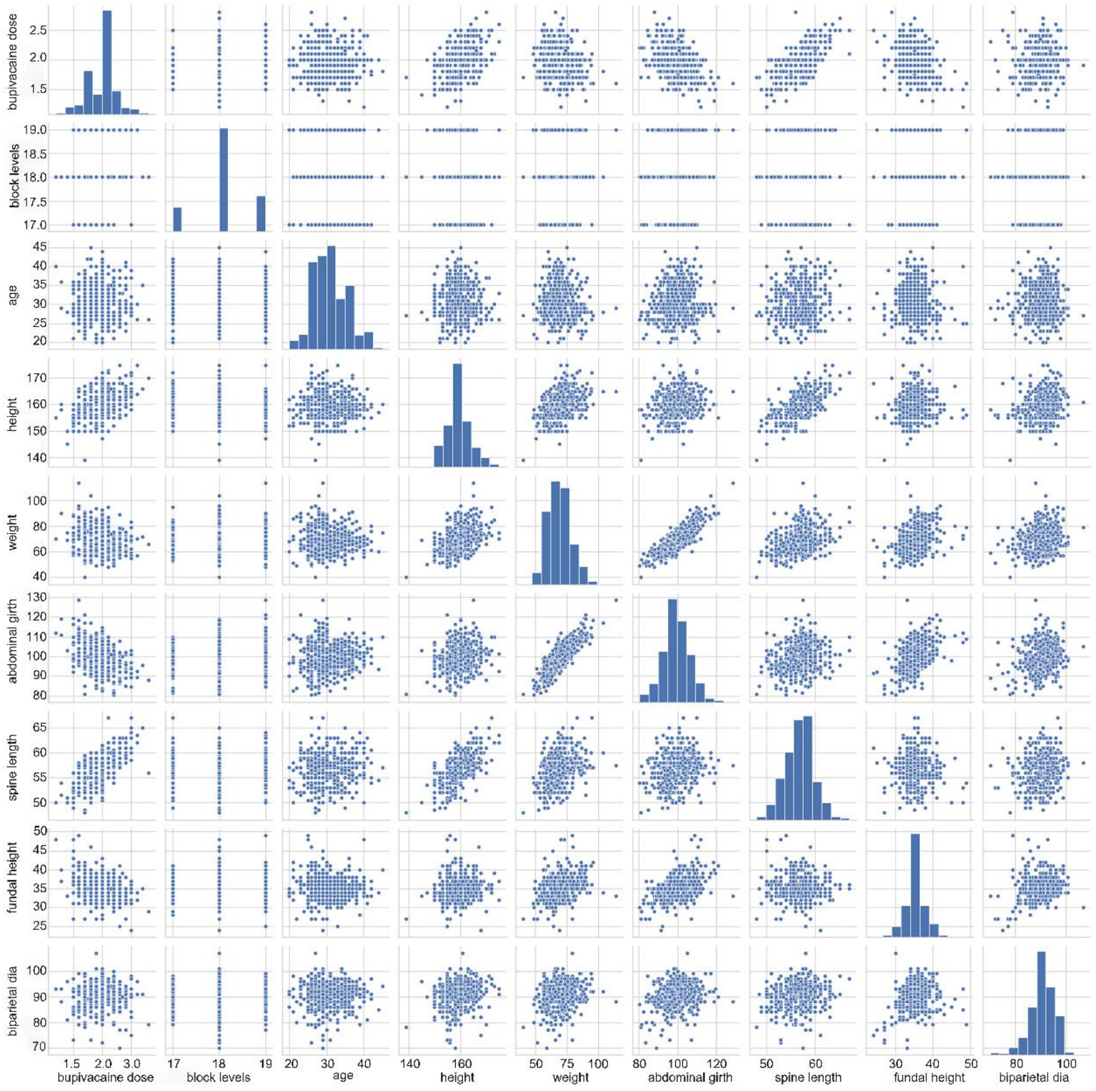

Fig 2. Correlation analysis diagram between physical variables and intrathecal bupivacaine dose for the parturients with block levels between T4 and T6.

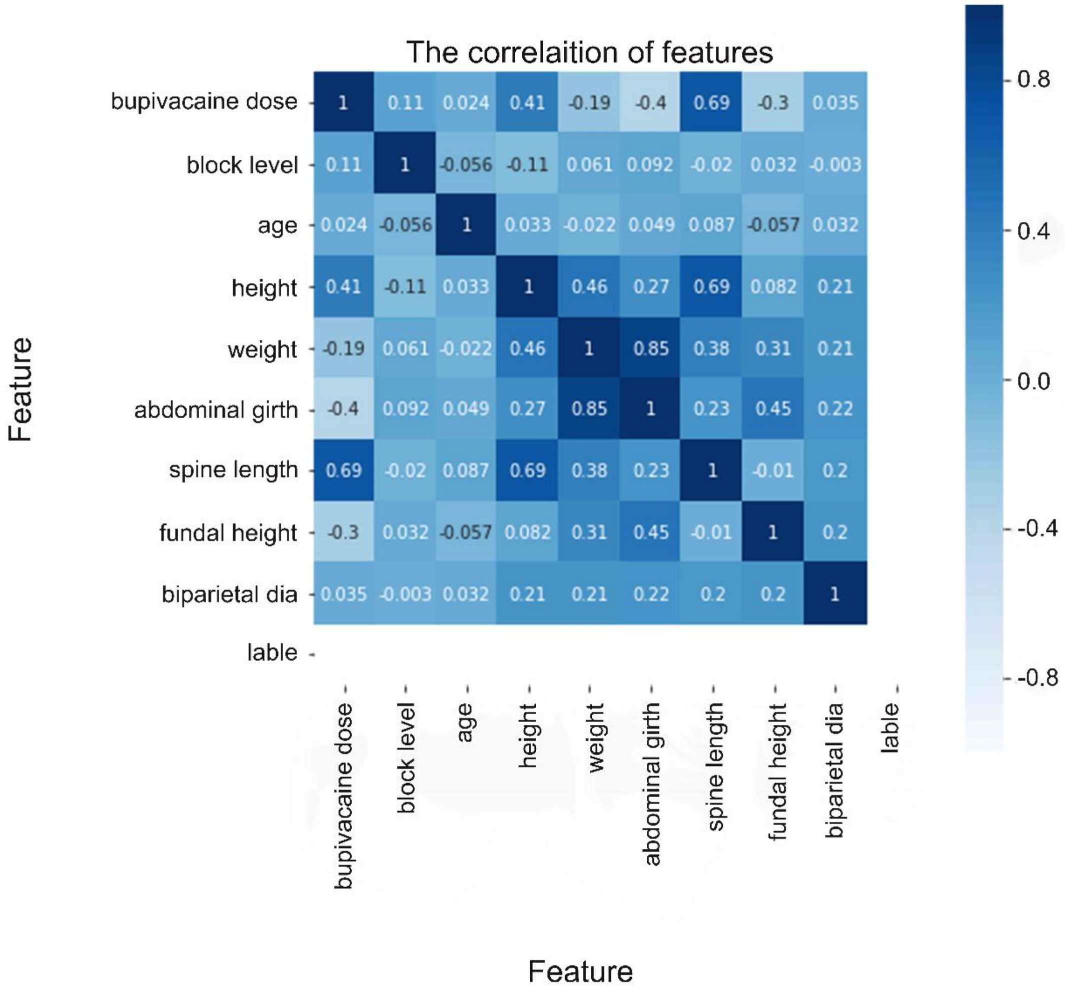

Supplement: Supplementary file 1 — Additional file 1: Figure S1. Visual analysis diagram of correlation between physical variables and intrathecal bupivacaine dose for the parturients with block levels between T4 and T6. Figure S2. Correlation analysis diagram between physical variables and intrathecal bupivacaine dose for the parturients with block levels between T4 and T6. [file 12871_2021_1331_MOESM1_ESM.pdf]
